# Black holes regulate cool gas accretion in massive galaxies

**DOI:** 10.1038/s41586-024-07821-2

**Published:** 2024-08-14

**Authors:** Tao Wang, Ke Xu, Yuxuan Wu, Yong Shi, David Elbaz, Luis C. Ho, Zhi-Yu Zhang, Qiusheng Gu, Yijun Wang, Chenggang Shu, Feng Yuan, Xiaoyang Xia, Kai Wang

**Affiliations:** 1 School of Astronomy and Space Science, Nanjing University, Nanjing, China; 2 Key Laboratory of Modern Astronomy and Astrophysics, Nanjing University, Ministry of Education, Nanjing, China; 3 Université Paris-Saclay, Université Paris Cité, CEA, CNRS, AIM, Gif-sur-Yvette, France; 4 Kavli Institute for Astronomy and Astrophysics, Peking University, Beijing, China; 5 Department of Astronomy, School of Physics, Peking University, Beijing, China; 6 Shanghai Key Lab for Astrophysics, Shanghai Normal University, Shanghai, China; 7 Center for Astronomy and Astrophysics and Department of Physics, Fudan University, Shanghai, China; 8 Tianjin Astrophysics Center, Tianjin Normal University, Tianjin, China

**Keywords:** Galaxies and clusters, Interstellar medium

## Abstract

The nucleus of almost all massive galaxies contains a supermassive black hole (BH)^1^. The feedback from the accretion of these BHs is often considered to have crucial roles in establishing the quiescence of massive galaxies^2–14^, although some recent studies show that even galaxies hosting the most active BHs do not exhibit a reduction in their molecular gas reservoirs or star formation rates^15–17^. Therefore, the influence of BHs on galaxy star formation remains highly debated and lacks direct evidence. Here, based on a large sample of nearby galaxies with measurements of masses of both BHs and atomic hydrogen (HI), the main component of the interstellar medium^18^, we show that the HI gas mass to stellar masses ratio (*μ*_HI_ = *M*_HI_/*M*_⋆_) is more strongly correlated with BH masses (*M*_BH_) than with any other galaxy parameters, including stellar mass, stellar mass surface density and bulge masses. Moreover, once the *μ*_HI_–*M*_BH_ correlation is considered, *μ*_HI_ loses dependence on other galactic parameters, demonstrating that *M*_BH_ serves as the primary driver of *μ*_HI_. These findings provide important evidence for how the accumulated energy from BH accretion regulates the cool gas content in galaxies, by ejecting interstellar medium gas and/or suppressing gas cooling from the circumgalactic medium.

## Abstract (web summary)

Supermassive black holes regulate the amount of atomic hydrogen in galaxies and the atomic hydrogen gas mass to stellar masses ratio is more strongly correlated with black hole masses.

## Main

Our primary sample consists of 69 central galaxies in the nearby Universe with direct estimates of black hole (BH) masses derived from resolved kinematics of stars or gas^11,19–21^. We have included only central galaxies to avoid any environmental impact on the interstellar medium (ISM) properties of galaxies. The sample includes several types of galaxy, including spirals, lenticulars and ellipticals. We obtained the atomic hydrogen (HI) 21-cm emission fluxes, which trace the atomic gas mass *M*_HI_, by crossmatching with nearby galaxy databases (Methods and Extended Data Table 1).

**Fig. 1:**
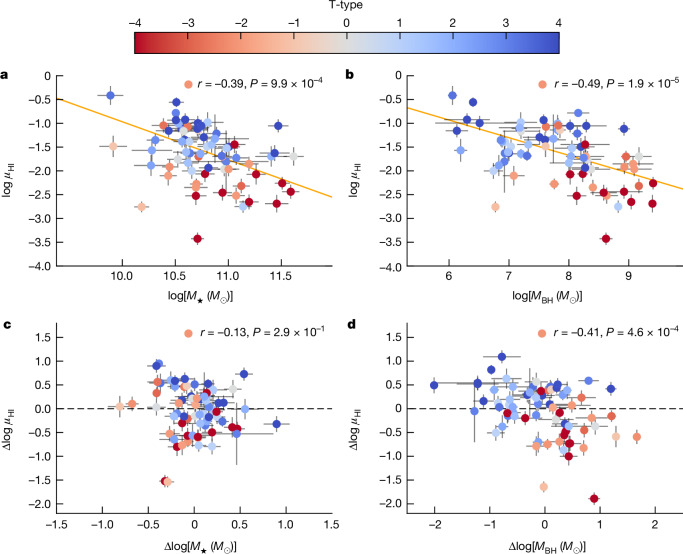
Comparison between the relations of *μ*_HI_ to *M*_⋆_ and *μ*_HI_ to *M*_BH_ for the BH sample. **a**,**b**, *μ*_HI_–*M*_⋆_ (**a**) and *μ*_HI_–*M*_BH_ (**b**) correlations. Galaxies are colour-coded by their morphological T types, with smaller values being more early-type and larger values more late-type morphologies. The orange lines represent the best-fitted linear relation, taking into account the uncertainties of both variables. **c**,**d**, Comparison of the partial correlation of *μ*_HI_*–M*_⋆_ (while controlling for *M*_BH_) (**c**) and *μ*_HI_*–M*_BH_ (while controlling for *M*_⋆_) (**d**). The *x-* and *y-*axes show the residual in *μ*_HI_ and *M*_⋆_ after removing their dependence on *M*_BH_ in the left panel, and *M*_BH_ after removing their dependence on *M*_⋆_ in the right panel: Δlog *μ*_HI_ = log *μ*_HI_ − log *μ*_HI_(*M*_BH_) and Δlog *M*_⋆_ = log *M*_⋆_ − log *M*_⋆_(*M*_BH_) in the left panel, and Δlog *μ*_HI_ = log *μ*_HI_ − log *μ*_HI_(*M*_⋆_) and Δlog *M*_BH_ = log *M*_BH_ − log *M*_BH_(*M*_⋆_) in the right panel. The horizontal dashed line indicates zero correlation, that is, there is no intrinsic correlation between the two quantities. The Spearman correlation coefficients between the two corresponding variables are shown in each panel. The error bars refer to 1*σ* measurement errors.

We define the HI gas content as the ratio of the HI mass and the stellar mass represented as *μ*_HI_ = *M*_HI_/*M*_⋆_. We first examine the relationship between *μ*_HI_ and BH masses and compare it with the *μ*_HI_–*M*_⋆_ correlation in Fig. 1. The correlation with *M*_BH_ is found to be more significant than the correlation with *M*_⋆_, with a Spearman correlation coefficient of *r* = −0.49 (*P* = 10^−4.7^) and *r* = −0.39 (*P* = 10^−3.0^), respectively. More importantly, the partial correlation between *μ*_HI_ and *M*_⋆_ while controlling for *M*_BH_, that is, removing both their dependence on *M*_BH_, indicates that *μ*_HI_ shows no dependence on *M*_⋆_ (*r* = −0.13, *P* = 0.29; Fig. 1 (bottom left)), whereas strong residual correlation exists between *μ*_HI_ and *M*_BH_ while controlling for *M*_⋆_ (*r* = −0.41, *P* = 10^−3.3^; Fig. 1 (bottom right)). Moreover, although the *μ*_HI_–*M*_⋆_ correlation differs significantly for early- and late-type galaxies with the early-type galaxies exhibiting systematically lower *μ*_HI_ at fixed *M*_⋆_, galaxies with different morphologies follow the same *μ*_HI_–*M*_BH_ relation. This suggests that the low HI values in those early-type galaxies on the *μ*_HI_*–M*_⋆_ relation are probably only a reflection that these galaxies have more massive BHs compared with late-type galaxies with similar *M*_⋆_.

**Extended Data Fig. 1:**
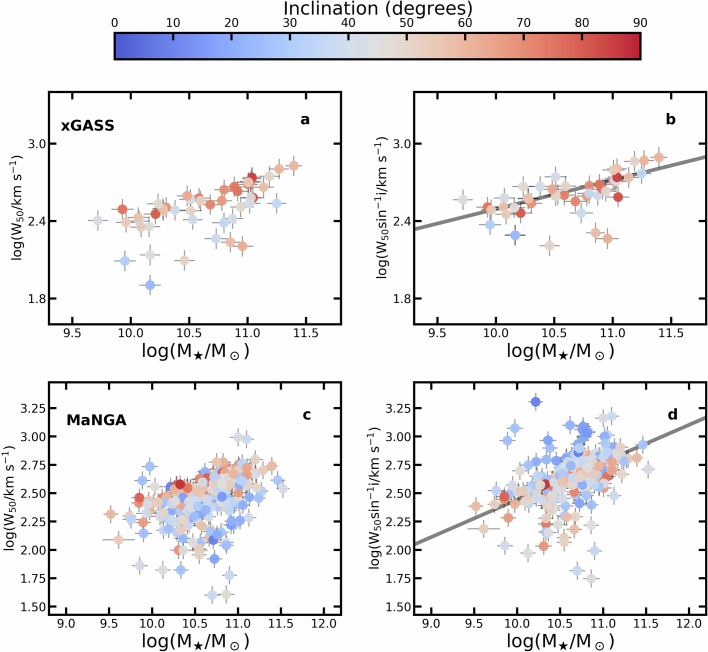
The impact of inclinations on the observed HI line width. The relation between *M*_⋆_ and the observed HI line width are shown in panels (**a, c**), while the relation between *M*_⋆_ and the inclination-corrected HI line width are shown in panels(**b, d**), for MaNGA and xGASS galaxies respectively. The error bars refer to 1-*σ* errors for log*M*_⋆_. For *W*_50_, we denote 20% measurement uncertainties.

Although the partial correlation between *μ*_HI_, *M*_BH_ and *M*_⋆_ offers direct evidence that BHs play a more crucial part than *M*_⋆_ in regulating *μ*_HI_, the heterogeneous nature of this sample makes it challenging to determine how the resulting relation could be applicable to broad galaxy populations. To validate this relation, we used a large sample of nearby galaxies with deep HI observations (Methods and Extended Data Fig. 1), which comprises 474 group central galaxies with 10^9.5^*M*_⊙_ < *M*_⋆_ < 10^11.5^*M*_⊙_ and reliable central velocity dispersion (*σ*) measurements. Out of this, 281 of them are detected in HI with HI upper limits available for the remaining 193 sources. *M*_BH_ for this galaxy sample is inferred from the *M*_BH_*–σ* relation (Methods). Hereafter we will call this enlarged galaxy sample ‘the galaxy sample’, and we call the sample with directly measured *M*_BH_ ‘the BH sample’.

**Fig. 2:**
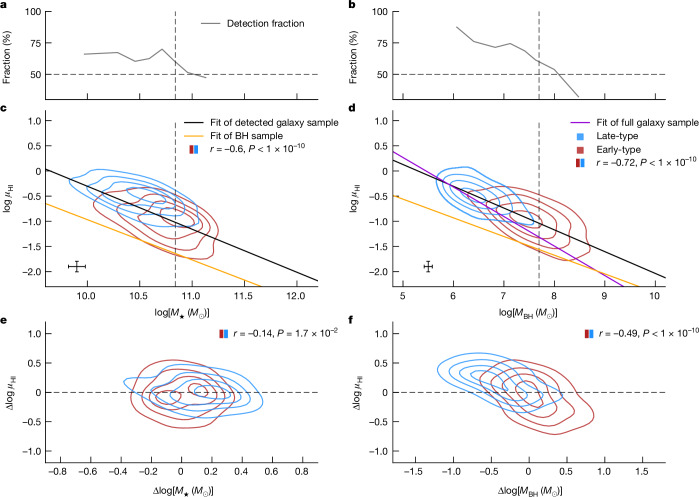
Comparison between the relations of *μ*_HI_ to *M*_⋆_ and *μ*_HI_ to *M*_BH_ for the galaxy sample. **a**,**b**, *μ*_HI_*–M*_⋆_ (**a**) and *μ*_HI_*–M*_BH_ (**b**) correlations. Galaxies are divided into early- and late-type galaxies based on their Sérsic indexes (separated at *n* = 2), which are shown in red and blue contours, respectively. The HI-detection rates of galaxies are shown as a function of stellar masses and BH masses. The vertical dashed lines indicate the position when the HI-detection fraction reaches 60%. **c**,**d**, *μ*_HI_*–M*_⋆_ (**c**) and *μ*_HI_*–M*_BH_ (**d**) relations. The best-fitted relations for the HI-detected galaxy sample and the BH sample are shown by the black and orange lines, respectively. We also show the *μ*_HI_*–M*_BH_ relation for the full galaxy sample with the magenta line in **d**. **e**,**f**, The partial correlation between *μ*_HI_ and *M*_⋆_ while controlling for *M*_BH_ (**e**), and the partial correlation between *μ*_HI_ and *M*_BH_ while controlling for *M*_⋆_ (**f**). The corresponding Spearman coefficients are shown in each panel. The median 1*σ* error bars for the galaxy sample are shown in **c** and **d**.

The *μ*_HI_*–M*_⋆_ and *μ*_HI_*–M*_BH_ relations for the galaxy sample are shown in Fig. 2. Both *M*_BH_ and *M*_⋆_ are found to be tightly correlated with *μ*_HI_ with respective *r* = −0.72 and *r* = −0.60. However, the partial correlation suggests that the *μ*_HI_*–M*_⋆_ correlation almost disappears (when controlling for *M*_BH_) with *r* = −0.14, compared with *r* = −0.49 for the *μ*_HI_*–M*_BH_ relation (when controlling for *M*_⋆_). This further suggests that the *μ*_HI_*–M*_⋆_ correlation is mostly driven by the *μ*_HI_*–M*_BH_ and *M*_⋆_*–M*_BH_ correlations. Similar to the BH sample, early- and late-type galaxies follow the same *μ*_HI_*–M*_BH_ relation, but a different *μ*_HI_*–M*_⋆_ relation.

The best-fitted *μ*_HI_–*M*_BH_ relation for the HI-detected galaxy sample yields a slope of −0.43 ± 0.02 (Fig. 2, black line), which is steeper than that for the BH sample (−0.37 ± 0.06; Fig. 2, orange line). This is most likely driven by the selection biases in the BH sample, which are more complete and representative at large *M*_BH_ but poorly sampled at low *M*_BH_. Moreover, we also derive an inherent *μ*_HI_–*M*_BH_ scaling relation (Fig. 2, magenta line) encompassing both HI detections and non-detections, resulting in a steeper slope (−0.59 ± 0.19) than that for fitting the HI detections exclusively (Extended Data Table 2).

**Fig. 3:**
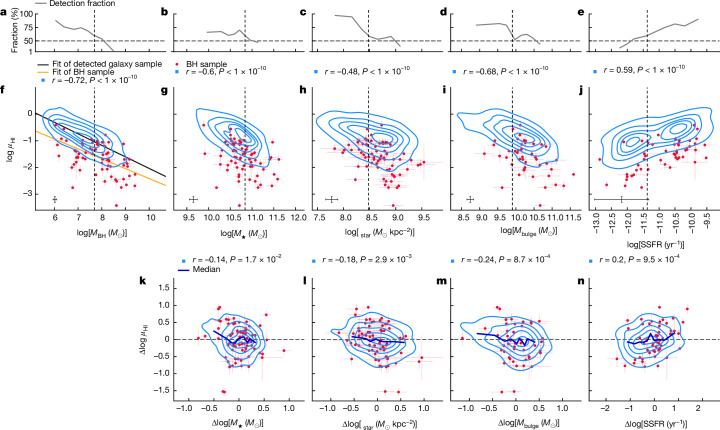
The impact of *M*_BH_ on the correlation between *μ*_HI_ and other main galactic parameters. **a**–**e**, The HI-detection fraction along *M*_BH_ (**a**) and some other main physical parameters of galaxies, including *M*_⋆_ (**b**), *Σ*_star_ (**c**), *M*_bulge_ (**d**) and SSFR (**e**). The vertical dashed lines indicate the position at which the HI-detection rates hit 60%. **f**–**j**, The relation between the parameters *M*_BH_ (**f**), *M*_⋆_ (**g**), *Σ*_star_ (**h**), *M*_bulge_ (**i**) and SSFR (**j**) and *μ*_HI_. The contours denote the distribution of the HI-detected galaxy sample, whereas the filled red circles denote the BH sample with 1*σ* error bars. The best-fitted *μ*_HI_–*M*_BH_ relations for the HI-detected galaxy sample and the BH sample are shown in **f** by the black and orange lines, respectively. The median 1*σ* error bars for the galaxy sample are shown. **k**–**n**, The relation between the residual in *μ*_HI_ and the residual in other galactic parameters after removing their dependence on *M*_BH_: Δlog *μ*_HI_ = log *μ*_HI_ − log *μ*_HI_(*M*_BH_) and Δlog *X* = log *X* − log *X*(*M*_BH_) with *X* representing *M*_⋆_ (**k**), *Σ*_star_ (**l**), *M*_bulge_ (**m**) and SSFR (**n**), and *μ*_HI_(*M*_BH_) and *X*(*M*_BH_) derived from their best-fitted relation with *M*_BH_ (Extended Data Fig. 2). The solid medium-blue lines in **k**–**n** show the running median of the residuals in *μ*_HI_. The Spearman correlation coefficients for the HI-detected galaxy sample between the corresponding *x* and *y* variables are shown in **f**–**n**.

**Extended Data Fig. 3:**
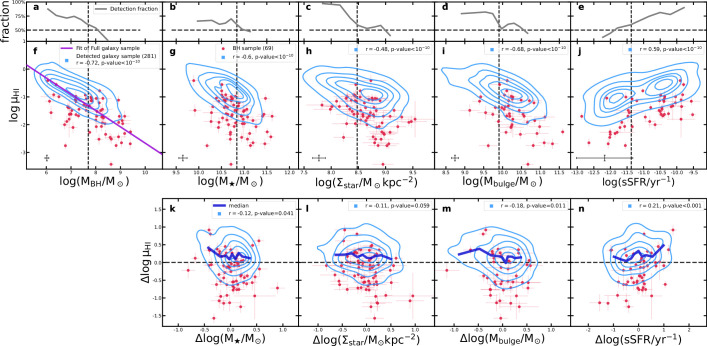
The impact of *M*_BH_ on the correlation between *μ*_HI_ and other major galactic parameters for the full galaxy sample. Similar as Fig. 3, but with the third row replaced by the relation between the residual in *μ*_HI_ and other galactic parameters after removing their dependence on *M*_BH_ based on the full sample instead of only the HI-detected ones. The solid medium-blue lines denote the running median of the residuals in *μ*_HI_. Since the HI-detected samples are always biased compared to the full sample, the running medians exhibits a positive offset compared to zero values(black dashed lines). The median error of the galaxy sample is shown in the lower left in panel (**f** ~ **j**). The error bars refer to 1-*σ* errors. In the top-right corners of the middle and bottom panels, we show the Spearman correlation coefficients for the HI-detected galaxy sample between the corresponding *x* and *y* variables.

Next, we explore further the correlations between *μ*_HI_ and other main galactic parameters^22^, including stellar surface densities (*Σ*_star_), bulge masses (*M*_bulge_) and specific star formation rates (SSFR), to determine whether *M*_BH_ is the key parameter in determining *μ*_HI_ in galaxies. Figure 3 compares the correlation among *μ*_HI_, *M*_⋆_, *M*_BH_, *Σ*_star_, *M*_bulge_ and SSFR for the HI-detected galaxy sample. Although significant correlations exist between *f*_HI_ and all these parameters, after removing their dependence on *M*_BH_, all the correlations almost disappear, with negligible Spearman coefficients and zero running medians. We also verify this by using the inherent *μ*_HI_–*M*_BH_ relation derived for the full sample in Extended Data Fig. 3, yielding consistent results.

Given that *M*_BH_, *M*_⋆_, *Σ*_star_ and *M*_bulge_ are all highly correlated, as a further test on the fundamental role of *M*_BH_ in driving the correlation with *μ*_HI_, we conduct a partial least squares regression between *f*_HI_ and the parameter set of *M*_BH_, *M*_⋆_, *Σ*_star_ and *M*_bulge_ for the HI-detected galaxy sample and the BH sample, which shows that *M*_BH_ is the most significant predictor parameter of *μ*_HI_ (Methods).

As *M*_BH_ is proportional to the integrated energy of BHs across their accretion history^2,13^, our findings offer observational evidence that the accumulated energy from BHs is vital in regulating the accretion and/or cooling of cool gas in galaxies. The immense energy released from the accretion of SMBHs in massive galaxies is known to be at least comparable to the binding energy of host galaxies^2,7,23^. This energy is thought to significantly affect the accretion of gas onto the galaxy and the cooling of the circumgalactic medium (CGM) and ISM. As *M*_⋆_ is closely linked with the inner halo binding energy (total binding energies within effective radii of galaxies)^24^, ${M}_{\star }\propto {E}_{{\rm{b}}}^{\beta }$, the *μ*_HI_–*M*_BH_ relation means ${M}_{{\rm{HI}}}\propto {M}_{\star }{M}_{{\rm{BH}}}^{-\alpha }\propto {E}_{{\rm{b}}}^{\beta }{M}_{{\rm{BH}}}^{-\alpha }$, where *E*_b_ represents the binding energy of the inner dark matter halo. At the stellar mass range probed by our BH sample, *β* ≈ 0.6, which is close to the value of *α*, yielding ${M}_{{\rm{HI}}}\propto {({E}_{{\rm{b}}}/{M}_{{\rm{BH}}})}^{\alpha }$ with *α* ≈ 0.6.

**Fig. 4:**
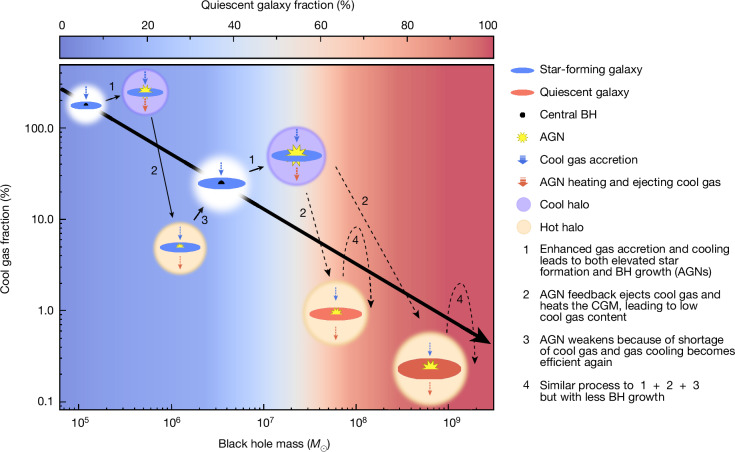
Schematic of the proposed scenario on how BHs regulate cool gas content in galaxies. The large arrow indicates the *μ*_HI_*–M*_BH_ correlation. The background colour scale indicates the quiescent galaxy fraction as a function of *M*_BH_, which shows a sharp increase at *M*_BH_ ≳ 10^7.5^*M*_⊙_ (Methods and Extended Data Fig. 5), corresponding to *μ*_HI_ < 10%. At fixed *M*_BH_, galaxies could maintain their *μ*_HI_ at a certain level determined by the relative strength of the inner halo binding energy and *M*_BH_. Once gas accretion is enhanced onto galaxies (and their BHs), which increases *μ*_HI_, *M*_BH_ will also grow and release additional heating energy that prevents further gas cooling or accretion. This will bring down *μ*_HI_ together with increasing *M*_⋆_ by star formation and reach a balance at higher *M*_BH_. Although the same process takes place in both SFGs and quiescent galaxies, the growth of *M*_BH_ or *M*_⋆_ should be much less significant in quiescent galaxies than in SFGs, and the large range of *M*_BH_ among quiescent galaxies (*M*_BH_ ~ 10^7−10^*M*_⊙_) is probably inherited from their different star-forming progenitors when they were quenched.

The analysis above indicates that the HI mass in galaxies is determined by the relative strength between the binding energy of the halo and the energy released from BHs (*E*_BH_ ∝ *M*_BH_). The binding energy of the halo determines how much gas can be accreted onto the dark matter halo, whereas the energy from BHs ejects or heats up the gas, preventing it from further cooling. The contest between the two determines how much accreted gas can be eventually cooled and settled down onto the central galaxies. For such a mechanism to be effective, a negative feedback loop involving gas accretion or cooling and BH accretion or feedback would be required^5,13^. The fact that the accreted cool gas could feed both star formation and BH accretion makes this possible. When gas accretion or cooling is elevated, stronger BH accretion is also triggered, resulting in more energy ejected into the ISM and CGM, which inhibits further cooling or accretion of the cool gas. This eventually brings down the cool gas content (and also the BH accretion rates). Conversely, a lower cool gas content would generally lead to weaker BH accretion with less energy ejection into the ISM and CGM, which will facilitate further cool gas accretion or cooling and increase *f*_HI_ until it reaches the average relation. The same physical process applies to both star-forming galaxies (SFGs) and quiescent galaxies. The difference is that although both *M*_BH_ and *M*_⋆_ of SFGs can grow substantially through this process, most quiescent galaxies will probably maintain their *M*_BH_ and *M*_⋆_ when they are quenched because of their overall low BH accretion rates and star formation rates. This scenario is shown in Fig. 4.

**Extended Data Fig. 4:**
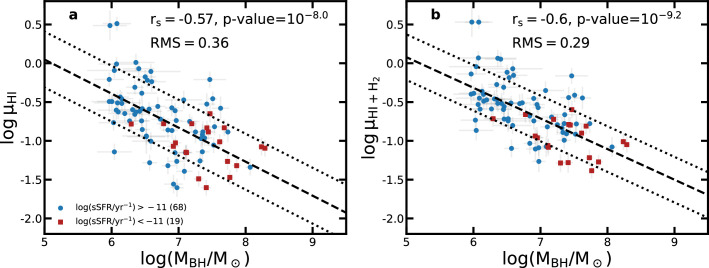
Comparison between the relations of BH masses to the HI gas fraction and to the total gas fraction. The relation between *M*_BH_ and HI gas fraction is shown in panel **a**, and the relation between *M*_BH_ and total gas fraction is shown in panel **b** for central galaxies. The blue circles and red squares denote respectively star-forming and quiescent galaxies, which are classified based on their SSFR. Spearman correlation coefficients and the RMS between data and the fitted relations (black dashed lines) are shown in the top-right corners. The error bars refer to 1-*σ* errors.

Under this scenario, the correlation between the total gas fraction (${\mu }_{{\rm{HI}}+{{\rm{H}}}_{2}}$) and *M*_BH_ is expected to be even tighter than the *μ*_HI_*–M*_BH_ relation. This is because gas cooling from the CGM will probably first cool as HI gas and only later become molecular gas that hosts star formation. In other words, HI gas probes only one phase of the cold gas, whereas AGN feedback should affect the cooling of both atomic and molecular gas in galaxies. This is probably the case. Although the sample with both HI and CO measurements is small, a reduced scatter for the ${\mu }_{{\rm{HI}}+{{\rm{H}}}_{2}}$–*M*_BH_ relation is found compared with the *μ*_HI_*–M*_BH_ relation (Methods and Extended Data Fig. 4). Apart from the small sample size, most of the galaxies with both HI and CO measurements are SFGs. Future studies with much larger and more representative galaxy samples with both HI and CO measurements will be needed to fully verify the ${\mu }_{{\rm{HI}}+{{\rm{H}}}_{2}}$–*M*_BH_ relation.

**Extended Data Fig. 5:**
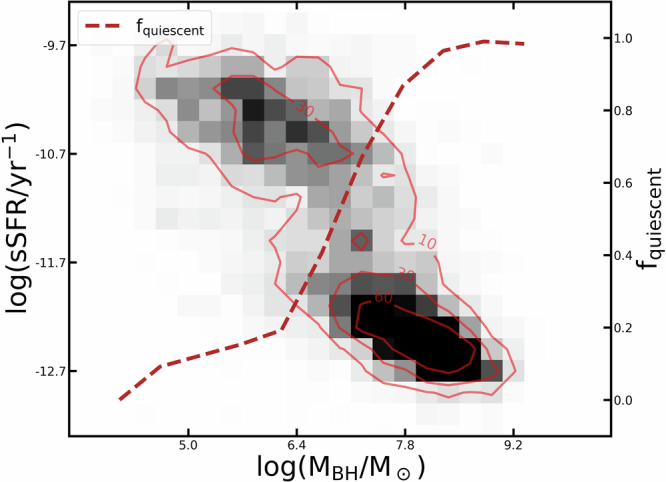
Relation between SSFR and BH masses for SDSS group central galaxies. Density map of SDSS group central galaxies is shown in the SSFR-*M*_BH_ plot. The dashed line denotes the quiescent fraction as a function of *M*_BH_. The contours and corresponding numbers denote the galaxy number for each pixel.

As cool gas is the material of star formation, these findings also shed critical light on the intimate connection between the presence of massive BHs and the quiescence of galaxies. It explains well why most quiescent galaxies are present only at *M*_BH_ ≳ 10^7.5^*M*_⊙_ (refs. ^10–13^) (Extended Data Fig. 5), corresponding to a low level of cool gas content (≲10%), hence minimal star formation rates. The proposed mechanism reconciles the discrepancy between the absence of strong instantaneous negative AGN feedback and the tight correlation between *M*_BH_ with galaxy quiescence. It is also consistent with empirical models indicating that the contest between dark matter halos and BHs governs the quenching of star formation in galaxies based on various observed galactic scaling relations^25^.

Although current studies have been confined to galaxies in the local Universe, the strong correlation across all redshifts between the quiescence of a galaxy and a prominent bulge, a high central stellar density or high central gravitational potential^26–30^, all of which suggest a large BH, implies that the same scenario may be applied to galaxies at high redshifts as well. Next-generation facilities, such as the Square Kilometer Array and the Next Generation Very Large Array, would be required to confirm this.

## Methods

### Cosmology

We adopted a Chabrier initial mass function (IMF)^31^ to estimate star formation rate and assumed cosmological parameters of *H*_0_ = 70 km s^−1^ Mpc^−1^, *Ω*_*M*_ = 0.3, and *Ω*_*Λ*_ = 0.7.

### Sample selection

#### The BH sample

The sample for galaxies with directly measured BH masses is primarily from ref. ^11^, which includes 91 central galaxies collected from refs. ^19–21^. We excluded 18 sources with BH masses measured with reverberation mapping and kept only those measured with dynamical methods. We then added another 63 galaxies with measured BH masses from recent literature, which were matched with the group catalogue^32^ of nearby galaxies to select only central galaxies. We obtained the HI flux densities and masses of this sample by crossmatching with the nearby galaxy database, HyperLeda^33^. Our final sample includes 69 central galaxies with 41 from ref. ^11^ and the remaining from the compilation of recent literature. In Extended Data Table 3, we list the basic properties of our BH sample.

#### The galaxy sample

The sample for galaxies with HI measurements and indirect BH mass measurements are from the extended GALEX Arecibo SDSS Survey (xGASS; ref. ^34^) and HI-MaNGA programme^35,36^, which include HI observations towards a representative sample of about 1,200 and 6,000 galaxies with 10^9^*M*_⊙_ < *M*_⋆_ < 10^11.5^*M*_⊙_, respectively. The depth of the survey also allows for stringent constraints on the upper limits for the HI non-detections, enabling a comprehensive assessment of *f*_HI_ for the entire sample. We limited the redshift z < 0.035 to ensure high HI-detection rates even at the highest stellar masses and BH masses. We selected only group central galaxies, which include at least one satellite galaxy in their groups, based on the crossmatch with the group catalogue^37–39^. Isolated central galaxies lacking any satellites in their groups are discarded because they may have probably suffered from additional environmental effects^40^. We derived the BH masses for the xGASS and HI-MaNGA samples with their velocity dispersion^21^ from SDSS DR17^37^ (*σ*_SDSS_, and we require *σ*_SDSS_ ≥ 70 km s^−1^):

1 $$\log \left(\frac{{M}_{{\rm{BH}}}}{{M}_{\odot }}\right)=(8.32\pm 0.04)+(5.35\pm 0.23)\log \left(\frac{{\sigma }_{{\rm{SDSS}}}}{200\,{\rm{km}}\,{{\rm{s}}}^{-1}}\right).$$

### Physical parameters of the BH and galaxy sample

#### Stellar masses

The stellar masses for the galaxy sample are taken from the MPA-JHU catalogue^41,42^, which are derived from SED fitting based on SDSS data. For the BH sample, because most of them lack the same photometric coverage as the galaxy sample, we derive their stellar masses from their K-band luminosity and velocity dispersion-dependent K-band mass-to-light ratio following ref. ^21^:

2 $${M}_{\star }/{L}_{{\rm{K}}}=0.1{\sigma }_{{\rm{e}}}^{0.45}.$$

As an accurate determination of *σ*_e_ is not available for all galaxies, we derived *σ*_e_ for the full BH sample from the tight correlation in ref. ^21^:

3 $$\begin{array}{l}\log \left(\frac{{\sigma }_{{\rm{e}}}}{{\rm{km}}}\,{{\rm{s}}}^{-1}\right)=(2.11\pm 0.01)+(0.71\pm 0.03)\log \left(\frac{{L}_{{\rm{K}}}}{1{0}^{11}{L}_{\odot }}\right)\\ \,\,\,\,\,\,\,+(-0.72\pm 0.05)\log \left(\frac{{R}_{{\rm{e}}}}{5\,{\rm{kpc}}}\right).\end{array}$$

**Extended Data Fig. 6:**
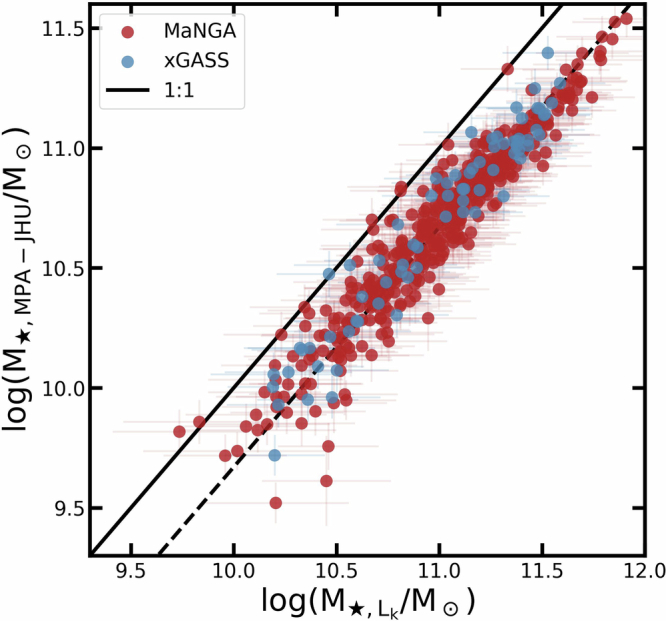
The comparison between the stellar masses derived from SED-fitting and those derived based on K-band luminosities. The SED-based estimation *M*_⋆_ is taken from the MPA-JHU catalog and the K-band luminosity based estimation of *M*_⋆_ is derived from the relation among *M*_⋆_, *L*_K_ and *R*_e_ given by Ref. ^21^. The dashed line denotes the ‘1:1’ line shifted by the mean mass difference of 0.32 dex. The error bars refer to 1-*σ* error.

To explore whether there are systematic differences between the two methods, we compare the stellar masses of the galaxy sample taken from the MPA-JHU catalogue and those derived from equation (2). A median mass difference 0.32 dex is found between the two methods (Extended Data Fig. 6), which may be attributed to the tilt from the fundamental plane beyond the mass-to-light ratio, for example, the dark matter component in the effective radius. We corrected these systematic mass differences for the BH sample to match that of the galaxy sample.

#### HI fraction and upper limits

The HI-detection limit depends not only on the sensitivity but also on the width of the HI line. To obtain more realistic upper limits, we first derived the expected HI line width for each HI non-detection. The width of the HI line indicates the circular velocity of the host galaxy, which should be proportional to the stellar masses. We explored this using the HI detections from the xGASS sample. Extended Data Fig. 1 shows the relation between *M*_⋆_ and the observed line width, as well as *M*_⋆_ and inclination-corrected line width. It indicates that the inclination-corrected line width is tightly correlated with *M*_⋆_, which is further used to derive the expected line width for the HI non-detections. Combining the sensitivity of the HI observations and the expected line width, we derived the upper limits for all the HI non-detections in our BH and galaxy samples.

#### Morphology

For BH sample, the morphology indicator *T* is obtained from the HyperLEDA database^33^. It can be a non-integer because for most objects the final *T* is averaged over various estimates available in the literature. For the galaxy sample, we classified them in to the early types and late types based on the Sérsic index (from NASA-Sloan Atlas catalogue; NSA: Blanton M.; http://www.nsatlas.org) larger or smaller than 2.

#### Star formation rates

The specific star formation rates (SSFR) of the galaxy sample are from the MPA-JHU catalogue based on ref. ^42^. The SSFR for the BH sample is taken from the original reference.

#### Bulge masses

The bulge information is from refs. ^43,44^ for the BH sample and galaxy sample, respectively. More specifically, we calculate the bulge mass for the galaxy sample using r-band *B*/*T*.

#### Stellar mass surface density

We calculated the K-band effective radius for both the BH and the galaxy sample according to ref. ^21^: log *R*_e_ = 1.16 log *R*_K_R_EFF_ + 0.23 log *q*_K_BA_, where *R*_e_ is the corrected apparent effective size, *R*_K_R_EFF_ and *q*_K_BA_ are K-band apparent effective radius and K-band axis ratio from 2MASS. After converting the apparent sizes to the physical sizes, the stellar mass surface density was derived as ${\varSigma }_{{\rm{star}}}={M}_{\star }/(2{\rm{\pi }}{R}_{{\rm{e}}}^{2})$.

#### H_2_ masses

We collected H_2_ masses from xCOLD GASS survey^18^ and ref. ^45^ for xGASS and MaNGA galaxies, respectively. We acknowledge that at least in the nearby Universe, the molecular-to-atomic gas mass ratio increases only weakly with stellar masses and remains relatively low over a wide stellar mass range, with $$R\equiv {M}_{{{\rm{H}}}_{2}}/{M}_{{\rm{HI}}} \sim 10-20 \% $$ at 10^9^*M*_⊙_ < *M*_⋆_ < 10^11.5^*M*_⊙_. We calculate the total gas fractions as $${\mu }_{{\rm{HI}}+{{\rm{H}}}_{2}}\,=$$$$({M}_{{\rm{HI}}}+{M}_{{{\rm{H}}}_{2}})/{M}_{\star }$$. For central galaxies (isolated centrals plus group centrals), we compare the *M*_BH_–*μ*_HI_ and *M*_BH_–$${\mu }_{{\rm{HI}}+{{\rm{H}}}_{2}}$$ relation in Extended Data Fig. 4. The *M*_BH_–$${\mu }_{{\rm{HI}}+{{\rm{H}}}_{2}}$$ relation exhibits a stronger correlation with the smaller scatter than the *M*_BH_*–μ*_HI_ relation. We acknowledge that, based on molecular hydrogen gas content traced through dust extinction, previous studies show an *M*_BH_–$${f}_{{{\rm{H}}}_{2}}$$ correlation^12^. Future studies with more direct measurements of molecular hydrogen gas for large samples will be needed to examine in detail whether *M*_BH_ also plays a fundamental part in regulating the molecular gas content in galaxies.

### Quiescent fraction

To estimate the quiescent fraction at different *M*_BH_, we selected galaxies from the MPA-JHU catalogue of SDSS galaxies with the same criteria as the galaxy sample, except that we limited the velocity dispersion to greater than 30 km s^−1^ to cover broader *M*_BH_ and we made no constraints on the HI detection. We classified the sample galaxies into star-forming and quiescent ones, separated at SSFR = −11. In each *M*_BH_ bin, the quiescent fraction was calculated as the ratio between the number of quiescent galaxies and that of all galaxies. The result is shown in Extended Data Fig. 5, which is consistent with that of previous work^29,46^.

### Linear least squares approximation

We implemented linear regression for the BH sample and the galaxy sample using Python package LTS_LINEFIT introduced in ref. ^47^, which is insensitive to outliers and can give the intrinsic scatter around the linear relation with corresponding errors of the fitted parameters.

### Linear fitting including upper limits

To incorporate both detections and upper limits in the galaxy sample, we applied the Kaplan–Meier non-parametric estimator to derive the cumulative distribution function at different *M*_BH_ bins (with Python package Reliability^48^), and performed 10,000 random draws from the cumulative distribution function at each bin to fit the relation between *f*_gas_ and *M*_BH_. The linear relation and its corresponding errors are taken as the best fitting and standard deviations of these fittings (Extended Data Table 2). The non-detection rate of HI is relatively low across most of the *M*_BH_ range and becomes significant only for galaxies with the most massive BHs (reaching about 50% at *M*_BH_ > 10^8^*M*_⊙_).

### Partial least square regression

To derive the most significant physical parameters in determining *μ*_HI_ statistically, we used the Python package Scikit-learn^49^ with partial least squares (PLS) Regression function, which uses a non-linear iterative partial least squares (NIPALS)^50^ algorithm. The PLS algorithm generalizes a few latent variables (or principal components) that summarize the variance of independent variables, which is used to find the fundamental relation between a set of independent and dependent variables. It has advantages in regression among highly correlated predictor variables. It calculates the linear combinations of the original predictor datasets (latent variables) and the response datasets with maximal covariance, then fits the regression between the projected datasets and returns the model:

4 $$Y=XB+F,$$

where *X* and *Y* are predictor and response datasets, *B* is the matrix of regression coefficients and *F* is the intercept matrix.

We constructed the *X* and *Y* matrices as the set of *M*_BH_, *M*_⋆_, *Σ*_star_, *M*_bulge_ and the set of *μ*_HI_. For the BH and galaxy samples, this returns the sample size of 45 and 189, respectively. The optimal number of latent variables (linear combinations of predictor variables) in PLS Regression is determined by the minimum of mean squared error from cross-validation (using function cross_val_predict in Scikit-learn) at each number of components. We find that the optimal number of latent variables for both the BH and the galaxy sample converges to one. Further increasing the number of latent variables yields only a few percentage changes in the mean squared errors, and *M*_BH_ remains the most significant predictor parameter. Following appendix B in ref. ^51^, the variance contribution from different parameters to *μ*_HI_ is decomposed as

5 $${\rm{Var}}(Y)=\mathop{\sum }\limits_{i=1}^{4}{\rm{Var}}({X}_{i}{B}_{i})+{\rm{Var}}(F),$$

where Var is a measure of the spread of a distribution. The portion of each parameter variance is shown in the last column of the Extended Data Table 3, which shows that *M*_BH_ dominates the variance. Further increasing the number of latent variables results only in a few percentage changes in the mean squared errors, and *M*_BH_ remains the most significant predictor parameter.

## Online content

Any methods, additional references, Nature Portfolio reporting summaries, source data, extended data, supplementary information, acknowledgements, peer review information; details of author contributions and competing interests; and statements of data and code availability are available at 10.1038/s41586-024-07821-2.

## Figures and Tables

**Extended Data Fig. 2:**
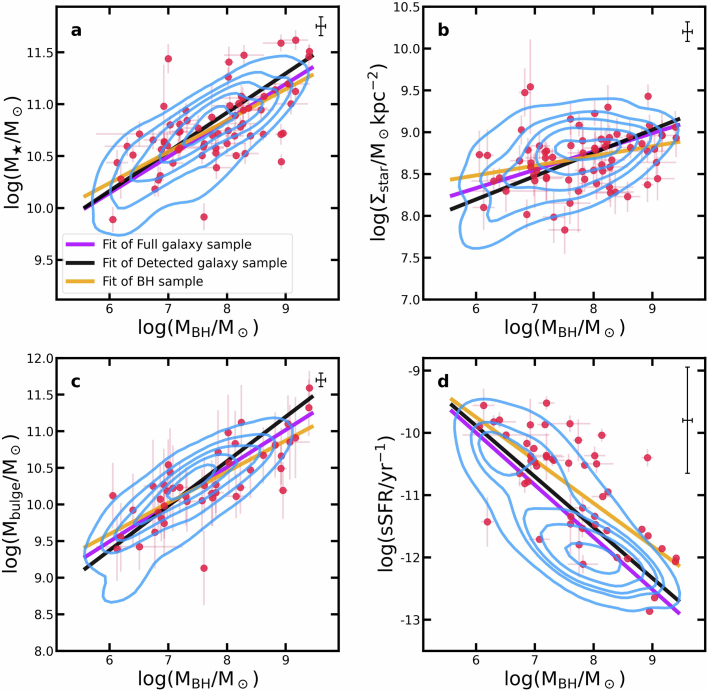
The relation between BH masses and other major galactic parameters for the BH and galaxy sample. The relation between *M*_BH_ and *M*_⋆_, *Σ*_star_, *M*_bulge_, and SSFR for the BH sample (red filled dots) and the full galaxy sample (blue contours) are shown in Panels **a**, **b**, **c**, and **d**, respectively. The best-fitted relation for the galaxy sample, the full galaxy, and the HI-detected galaxy sample are respectively drawn in solid orange, darkviolet, and black lines in each panel. These relations are used to derive the residuals of the corresponding galactic parameters after controlling for *M*_BH_ in the bottom row of Fig. 3, and Extended Data Fig. 3. The median error of the galaxy sample is shown in the upper right in each panel. The error bars refer to 1-*σ* errors.

**Extended Data Table 1:**
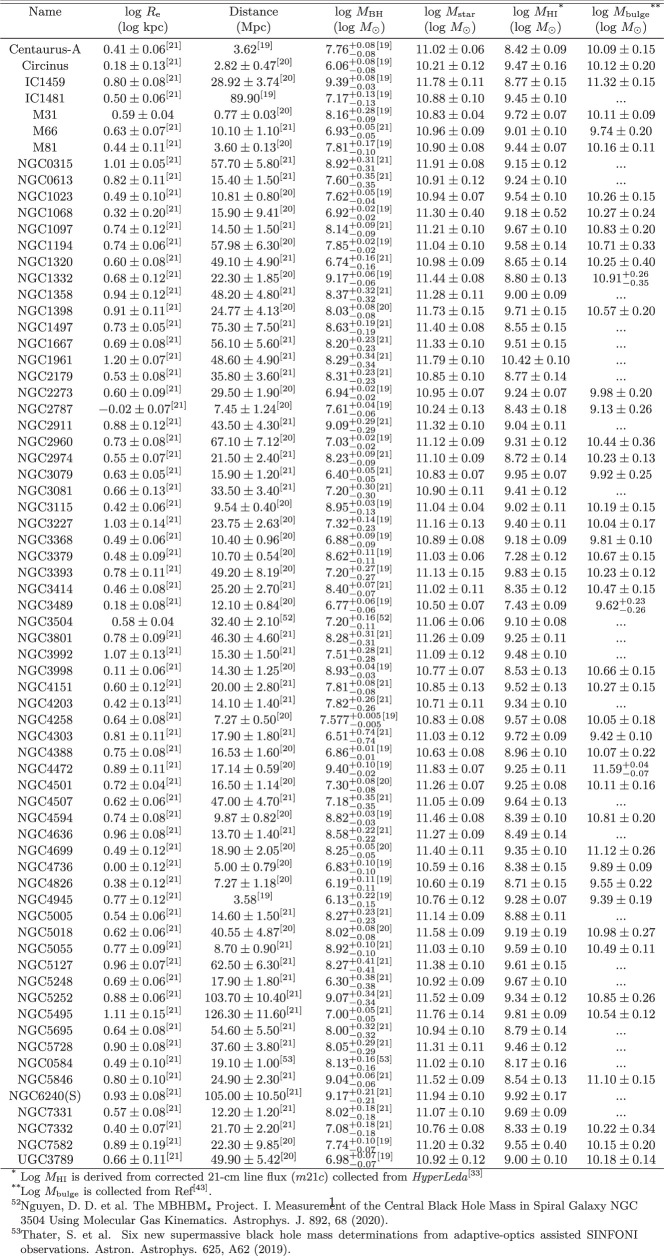
BH sample properties

**Extended Data Table 2:**
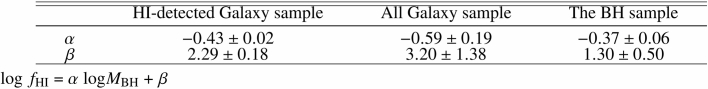
Best-fitted linear relations between *μ*_HI_ and *M*_BH_ for different samples

**Extended Data Table 3:**
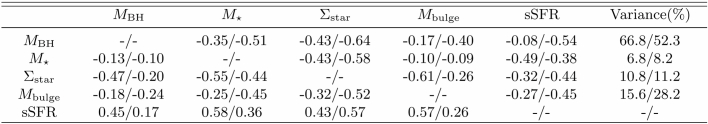
Partial correlation coefficients and variance contributions regarding the correlation between f_HI_ and major galactic parameters

## Data Availability

All data used in this paper are publicly available, and the key physical parameters of the BH sample are summarized in Extended Data Table 1.

## Code availability

All codes used in the paper are publicly available.
